# Diagnosis and Multimodality Management of Cushing's Disease: A Practical Review

**DOI:** 10.1155/2013/893781

**Published:** 2013-01-15

**Authors:** Gabriel Zada

**Affiliations:** Department of Neurosurgery, Keck School of Medicine of USC, 1200 North State Street, Suite 3300, Los Angeles, CA 90089, USA

## Abstract

Cushing's Disease is caused by oversecretion of ACTH from a pituitary adenoma and results in subsequent elevations of systemic cortisol, ultimately contributing to reduced patient survival. The diagnosis of Cushing's Disease frequently involves a stepwise approach including clinical, laboratory, neuroimaging, and sometimes interventional radiology techniques, often mandating multidisciplinary collaboration from numerous specialty practitioners. Pituitary microadenomas that do not appear on designated pituitary MRI or dynamic contrast protocols may pose a particularly challenging subset of this disease. The treatment of Cushing's Disease typically involves transsphenoidal surgical resection of the pituitary adenoma as a first-line option, yet may require the addition of adjunctive measures such as stereotactic radiosurgery or medical management to achieve normalization of serum cortisol levels. Vigilant long-term serial endocrine monitoring of patients is imperative in order to detect any recurrence that may occur, even years following initial remission. In this paper, a stepwise approach to the diagnosis, and various management strategies and associated outcomes in patients with Cushing's Disease are discussed.

## 1. Introduction

Cushing's Disease is a life-threatening illness defined by the chronic excess of serum cortisol in the presence of an ACTH-secreting pituitary adenoma and accounts for approximately 80% of newly diagnosed cases of Cushing's syndrome (excess systemic cortisol from any source). Patients with ACTH oversecretion from a pituitary adenoma may present with Cushing's Disease or Nelson's syndrome, depending on the functionality of the adrenal glands. Functional ACTH-staining adenomas comprise approximately 14% of all surgically resected pituitary adenomas [1–3]. Cushing's Disease is typically diagnosed during the third and fourth decades of life and occurs eight times more commonly in women than men [4]. The disease may also manifest in children and adolescents and comprises a larger proportion of all pituitary adenoma subtypes in pediatric patients as compared to adults [5, 6]. If left untreated, an ACTH-adenoma often results in diminished patient survival and worsened quality of life, due to its severe effects on several physiological systems of the body [7–10].

## 2. Clinical Presentation of Cushing's Disease

The typical clinical symptoms and physical characteristics in patients with Cushing's Disease include acne, hirsutism/hair loss, weight gain, lipodystrophy, moon facies, skin bruising, abdominal striae, insomnia, and amenorrhea. Medical conditions associated with Cushing's Disease include diabetes mellitus, hypertension, osteoporosis, and arthralgia, among others. Furthermore, many psychological disturbances, including anxiety, depression, insomnia, psychosis, euphoria, and short-term memory/cognitive deficits, occur commonly in patients with Cushing's Disease.

Nelson's syndrome occurs in patients with ACTH-secreting adenomas that have undergone bilateral adrenalectomy and subsequently go on to develop excess serum levels of CRH and ACTH, typically developing 1–4 years later [11, 12]. The classical presentation of Nelson's syndrome includes characteristic bronzing of the skin (due to proopiomelanocortin expression), frequent enlargement of the residual pituitary adenoma due to loss of negative feedback inhibition, and elevated serum ACTH levels (typically greater than 200 ng/L) [11]. Hyperpigmentation commonly occurs on the extensor surfaces, knuckles, gingivae, scars, and areola. In modern series, however, hyperpigmentation occurs in only 42% of patients, likely due to improved surveillance techniques with laboratory and imaging studies [13]. Because of improvements in the diagnosis and management of ACTH-secreting tumors, and more stringent indications for performing bilateral adrenalectomies, Nelson's syndrome has become a relatively uncommon entity [14, 15].

## 3. Diagnosis of Cushing's Syndrome and Disease

**Figure 1:**
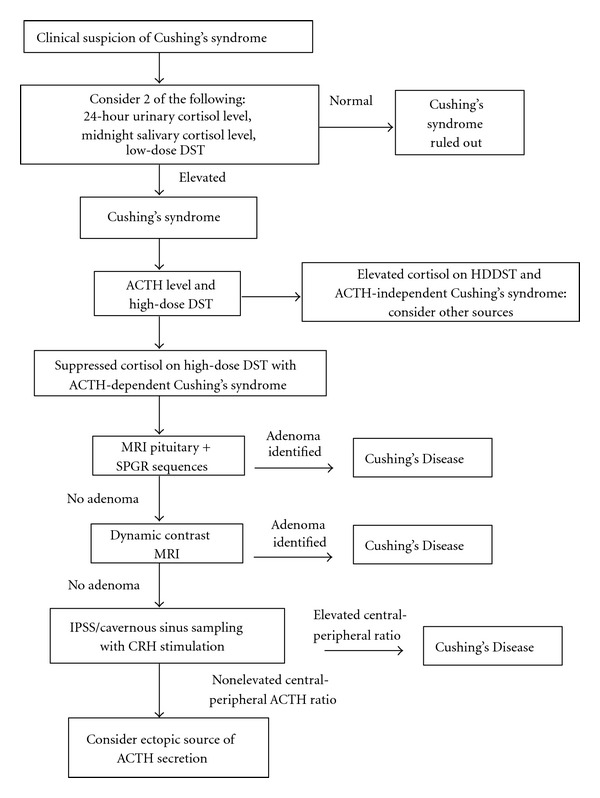
A stepwise algorithm for the diagnosis of Cushing's Disease. (Abbreviations: ACTH: adrenocorticotropic hormone, DST: dexamethasone suppression test, MRI: magnetic resonance imaging, SPGR: spoiled gradient recall, CRH: corticotropin-releasing hormone, IPSS: inferior petrosal sinus sampling).

Establishing an accurate diagnosis of Cushing's Disease relies on a thorough and stepwise sequence of laboratory and imaging studies (Figure 1) [16]. If clinical suspicion for Cushing's Syndrome exists, one of several screening tests for hypercortisolism should be performed, including a night-time salivary cortisol test, a 24-hour urinary-free cortisol test, a 1 mg overnight dexamethasone suppression test (DST), or a longer low-dose DST (0.5 mg every 6 hours for 48 hours) [16]. A second test for hypercortisolemia is preferable to confirm a diagnosis of Cushing's syndrome, followed by a serum ACTH level to differentiate ACTH-dependent from ACTH-independent hypercortisolemia. 

In patients with ACTH-dependent Cushing's syndrome (a majority), an MRI of the sella with contrast administration should be performed next. MRI may be negative in as many as 40% of cases of Cushing's Disease, despite the presence of a pituitary ACTH microadenoma, and additional modalities may therefore be required to establish the diagnosis. Among patients with Cushing's Disease and a pituitary adenoma identified on MRI, 85–87% have microadenomas (tumor diameter <10 mm) and the remaining 13–15% have macroadenomas (diameter ≥10 mm) [2, 17]. Invasion of surrounding regions occurs in 13–25% of cases, and is more common in patients with Nelson's syndrome [17]. ACTH-adenomas are typically hypoenhancing on T1 imaging following contrast administration and may be hyperintense on T2 imaging as compared to the normal pituitary gland [18]. Dynamic contrast MRI has been reported to provide a diagnostic advantage for selected cases of small microadenomas and is recommended if standard pituitary MR imaging is negative [19]. Spoiled-gradient recall acquisition with thin-slice imaging has also been reported to substantially improve imaging resolution and the diagnosis of small microadenomas [20].

If MR imaging is negative, yet a strong suspicion for Cushing's Disease exists, a high-dose dexamethasone suppression test and/or inferior petrosal sinus sampling (IPSS) may be performed. During an IPSS test, serial endovascular venous blood sampling for measuring ACTH is performed from the inferior petrosal and cavernous sinuses and peripheral venous blood following administration of corticotrophin-releasing hormone (CRH), which allows differentiation of Cushing's Disease from ectopic ACTH secretion. IPSS provides a sensitivity and specificity of 92–100% for the diagnosis of an ACTH microadenoma [21–23] and has been reported to accurately predict the laterality of the microadenoma in 60–84% of patients if one side demonstrates a measured ACTH level 1.4 times higher than the contralateral side [24].

## 4. Pathology of ACTH Adenomas

Hormonally active ACTH-staining adenomas comprise approximately 14% of all pituitary tumors resected via transsphenoidal operations [1–3]. In addition to demonstrating positive immunoreactivity for ACTH, these tumors frequently stain for the Periodic Acid-Schiff (PAS) immunomarker. In addition, ACTH adenomas may be densely granulated (50%) or sparsely granulated (50%) [2]: Densely granulated (basophilic) ACTH adenomas are strongly PAS and CAM5.2 (stains intracellular keratin) positive. Sparsely granulated (chromophobic) ACTH adenomas, on the other hand, are weakly PAS positive, have more frequent cellular pleomorphism, and are typically more aggressive adenomas. Silent-ACTH adenomas are a separate entity characterized by aggressive, nonfunctional tumors with positive ACTH immunoreactivity, and no clinical evidence of Cushing's Disease or Nelson's Syndrome [25]. 

Crooke's hyaline change refers to a reactive process in adenohypophyseal cells in the setting of chronic hypercortisolemia. The presence of Crooke's hyaline change, without an adenoma, has been reported in surgical specimens from 1.8% of transsphenoidal operations and in some cases may be associated with intraoperative loss of a microadenoma specimen [2]. Crooke's cell adenoma refers to a pituitary adenoma arising from reactive adenohypophyseal cells with Crooke's hyaline change and has been reported to account for less than 1% of pituitary adenomas [2].

## 5. Clinical Management of Cushing's Disease

**Figure 2:**
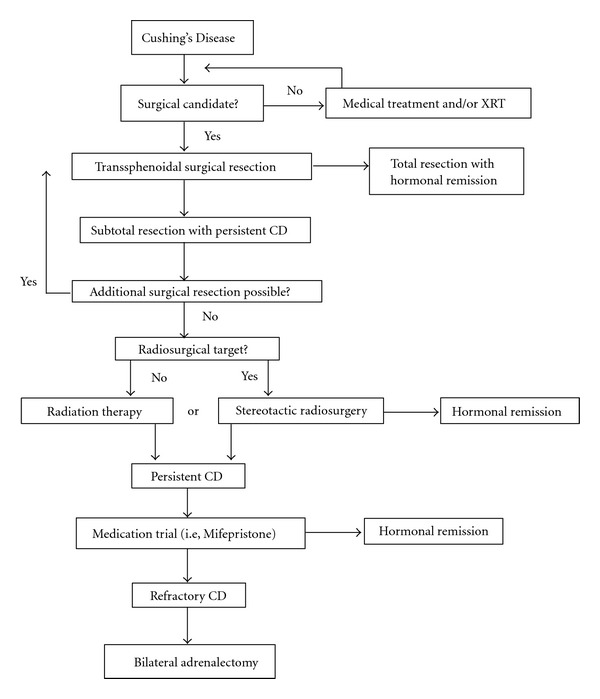
A stepwise algorithm for the multimodality treatment options available in Cushing's Disease. (Abbreviations: XRT: radiation therapy, CD: Cushing's Disease).

Because ACTH adenomas are typically microadenomas that do not cause a substantial degree of mass effect on surrounding structures, the primary goal in treating a majority of patients with Cushing's Disease is establishing endocrinological remission, as defined by normalization (or preferably subnormal levels) of serum and urine cortisol (Figure 2). Surgery remains the primary option for most patients with a new diagnosis of Cushing's Disease [26]. Following the attempted surgical management of Cushing's Disease, multimodal therapy may be indicated for patients with persistent hypercortisolemia that do not achieve hormonal remission [26–28]. Adjunctive treatments following surgical management may include medical therapy, radiosurgery or other radiation-based treatments, and/or bilateral adrenalectomy. Despite improvements in overall survival and quality of life following multimodality treatment and achievement of hormonal remission, many patients with treated Cushing's Disease continue to have persistently diminished quality of life, as compared to normal age- and sex-matched controls [10, 29, 30].

## 6. Surgical Management of Cushing's Disease

Because medical management in patients with Cushing's Disease remains limited and carries a significant risk of systemic toxicity, surgical resection provides the best overall outcomes available to patients at this time. The transsphenoidal operation for patients with ACTH adenomas is the gold standard procedure, with excellent outcomes and low surgical risks reported in a majority of patients, especially those with microadenomas [31]. Postoperative remission following surgical resection of ACTH-adenomas is generally achieved between 70 and 90% of patients and depends on tumor size and degree of invasion into surrounding regions [3, 15, 27, 31–33]. In recent years, endoscopic techniques have provided improved intraoperative visualization, which is especially beneficial in cases for small microadenomas and in MRI-negative Cushing's Disease, where microdissection and preservation of the normal pituitary gland are of paramount importance. Extracapsular resection of pituitary microadenomas should be attempted if a pseudocapsule exists and the tumor can be dissected from the normal gland in an en bloc fashion [34]. During surgery, a wide exposure of the entire sella and pituitary gland is routinely performed, even in cases of lateralized microadenomas, in order to survey the entire gland. Following tumor resection, the remainder of the gland is inspected thoroughly for evidence of additional tumors, as multiple ACTH adenomas have been reported in patients with Cushing's Disease [35].

## 7. Postoperative Surveillance in Patients with Cushing's Disease

During the first several days following surgical resection of an ACTH adenoma, frequent (i.e., every 6 hours) cortisol levels are measured until a nadir cortisol level is reached (optimally less than 2 *μ*g/dL) [36]. Replacement therapy (i.e., hydrocortisone) may be initiated at this time, especially if the patient develops clinical symptoms suggestive of cortisol withdrawal, such as headache, nausea/vomiting, and fatigue [26]. In symptomatic patients with nausea and vomiting, parenteral (intravenous) hydrocortisone replacement may be transiently required if oral medications are not tolerated.

The gold standard tests for assessment of endocrinological remission are a delayed morning fasting serum cortisol test and a 24-hour UFC test. In Nelson's syndrome, a normalized ACTH level defines endocrinological remission [13]. Rates of remission following transsphenoidal surgery for ACTH-producing tumors have been reported in 70–90% of patients with noninvasive microadenomas, and approximately 25–50% in patients with invasive macroadenomas [3, 15, 31, 37]. In patients with Cushing's Disease who do not reach subnormal levels following surgery, immediate reoperation and gland reexploration should be considered, especially if histopathology is negative for tumor. In patients without early remission, early reoperation within 60 days, usually for hemihypophysectomy or total hypophysectomy, has resulted in remission in 38–67% of patients [15, 38]. It should also be noted that delayed endocrinological remission, with a mean time to remission of 38 days, has been reported in 5.6% of patients following surgery, yet has also been associated with higher recurrence rates [39]. In patients achieving initial remission, recurrence has been reported to occur in approximately 17–25%, with a median time to recurrence of 3–5 years [1]. Some tumors may recur over a decade following initial remission, however, warranting long-term serial endocrine monitoring [1, 15].

## 8. Medical Management of Cushing's Disease

Medical therapy for Cushing's Disease remains limited, with the most effective intervention for this condition being surgery, as described above. Adrenal steroid synthesis inhibitors, including ketoconazole, have been successfully used to reduce serum cortisol levels in up to 70% of patients [40, 41]. Significant side effects, however, including gastrointestinal distress, gynecomastia, decreased libido, and impotence have been associated with this medication and other inhibitors of adrenal steroid synthesis. In addition, hepatotoxicity has been reported in up to 12% of patients, mandating frequent liver function laboratory assessments [40]. Second-line adrenal enzyme inhibitors, including metyrapone and mitotane, are also available for refractory Cushing's Disease, yet also carry substantial systemic risk profiles. The dopamine agonist bromocriptine has been attempted for ACTH-secreting adenomas with limited success rates [41]. Cortisol receptor antagonists, such as mifepristone (RU-486), are currently being evaluated as potential medical agents for Cushing's Disease and may play a first-line role in the treatment of Cushing's Disease in the years to come [42]. 

## 9. Stereotactic Radiosurgery

External beam radiation has been utilized in the past for refractory Cushing's Disease, although it carries a significant risk profile with regard to visual loss and hypopituitarism and has been replaced by focused stereotactic radiosurgery in the majority of centers [43]. Stereotactic fractionated or single-session radiosurgery is commonly utilized as an adjunctive treatment for residual disease, with typical marginal isodose levels of 18–25 Gy, depending on the tumor volume and proximity to surrounding structures. This modality is especially useful for tumors with residual cavernous sinus disease that is not surgically accessible. Hormonal remission has been reported in up to 54% following Gamma Knife radiosurgery; however, remission may take one to two years to achieve following radiosurgical treatment [31, 44, 45].

## 10. Bilateral Adrenalectomy

Bilateral adrenalectomy remains a viable option for patients with refractory or progressive Cushing's Disease and is typically used as a last resort option for patients with refractory disease following surgical, medical, and radiosurgical options. Remission from Cushing's Disease has been reported in 80–100% of patients following bilateral adrenalectomy [46]. Furthermore, improved quality of life has been reported in up to 89% of patients following bilateral adrenalectomy for Cushing's Disease, with undetectable cortisol levels measured in almost 80% of patients [46]. Nelson's syndrome has been reported to develop in approximately 8–30% of patients and can be associated with aggressive, invasive lesions, requiring serial imaging and laboratory monitoring of ACTH levels [12, 13].

## 11. Conclusions

Cushing's Disease is a life-threatening disorder caused by ACTH hypersecretion from a pituitary adenoma. An accurate and thorough stepwise diagnostic workup is essential for all patients prior to recommending surgical intervention. Transsphenoidal surgery remains the most reliable option for achieving hormonal remission, especially in patients harboring ACTH microadenomas. MRI-negative microadenomas may present a challenging situation and often require additional diagnostic testing such as IPSS, and intraoperative techniques including endoscopy and thorough gland exploration. Adjunctive interdisciplinary management is often required to achieve hormonal remission in many patients and frequently consists of medical and/or radiosurgical treatment in addition to surgery. Vigilant long-term serial endocrine monitoring of patients is imperative in order to detect any recurrence that may occur, even years following an initial remission.

## References

[1] Patil CG, Prevedello DM, Lad SP (2008). Late recurrences of Cushing’s disease after initial successful transsphenoidal surgery. *Journal of Clinical Endocrinology and Metabolism*.

[2] Saeger W, Lüdecke DK, Buchfelder M, Fahlbusch R, Quabbe HJ, Petersenn S (2007). Pathohistological classification of pituitary tumors: 10 years of experience with the German Pituitary Tumor Registry. *European Journal of Endocrinology*.

[3] Zada G, Kelly DF, Cohan P, Wang C, Swerdloff R (2003). Endonasal transsphenoidal approach for pituitary adenomas and other sellar lesions: an assessment of efficacy, safety, and patient impressions. *Journal of Neurosurgery*.

[4] Mindermann T, Wilson CB (1994). Age-related and gender-related occurrence of pituitary adenomas. *Clinical Endocrinology*.

[5] Kanter AS, Diallo AO, Jane JA (2005). Single-center experience with pediatric Cushing’s disease. *Journal of Neurosurgery*.

[6] Savage MO, Storr HL, Chan LF, Grossman AB (2007). Diagnosis and treatment of pediatric Cushing’s disease. *Pituitary*.

[7] Clayton RN, Raskauskiene D, Reulen RC, Jones PW (2011). Mortality and morbidity in Cushing’s disease over 50 Years in Stoke-on-Trent, UK: audit and meta-analysis of literature. *Journal of Clinical Endocrinology and Metabolism*.

[8] Dekkers OM, Biermasz NR, Pereira AM (2007). Mortality in patients treated for Cushing’s disease is increased, compared with patients treated for nonfunctioning pituitary macroadenoma. *Journal of Clinical Endocrinology and Metabolism*.

[9] Feelders RA, Pulgar SJ, Kempel A, Pereira AM (2012). The burden of Cushing's disease (CD): clinical and health-related quality of life aspects. *European Journal of Endocrinology*.

[10] van Aken MO, Pereira AM, Biermasz NR (2005). Quality of life in patients after long-term biochemical cure of cushing’s disease. *Journal of Clinical Endocrinology and Metabolism*.

[11] Banasiak MJ, Malek AR (2007). Nelson syndrome: comprehensive review of pathophysiology, diagnosis, and management. *Neurosurgical Focus*.

[12] Gil-Cárdenas A, Herrera MF, Díaz-Polanco A, Rios JM, Pantoja JP (2007). Nelson’s syndrome after bilateral adrenalectomy for Cushing’s disease. *Surgery*.

[13] Kelly PA, Samandouras G, Grossman AB, Afshar F, Besser GM, Jenkins PJ (2002). Neurosurgical treatment of Nelson’s syndrome. *Journal of Clinical Endocrinology and Metabolism*.

[14] Hornyak M, Weiss MH, Nelson DH, Couldwell WT (2007). Nelson syndrome: historical perspectives and current concepts. *Neurosurgical Focus*.

[15] Kelly DF (2007). Transsphenoidal surgery for Cushing’s disease: a review of success rates, remission predictors, management of failed surgery, and Nelson’s Syndrome. *Neurosurgical Focus*.

[16] Nieman LK, Biller BMK, Findling JW (2008). The diagnosis of Cushing’s syndrome: an endocrine society clinical practice guideline. *Journal of Clinical Endocrinology and Metabolism*.

[17] Scheithauer BW, Kovacs KT, Laws ER, Randall RV (1986). Pathology of invasive pituitary tumors with special reference to functional classification. *Journal of Neurosurgery*.

[18] Jagannathan J, Sheehan JP, Jane JA (2007). Evaluation and management of Cushing syndrome in cases of negative sellar magnetic resonance imaging. *Neurosurgical Focus*.

[19] Kucharczyk W, Bishop JE, Plewes DB, Keller MA, George S (1994). Detection of pituitary microadenomas: comparison of dynamic keyhole fast spin-echo, unenhanced, and conventional contrast-enhanced MR imaging. *American Journal of Roentgenology*.

[20] Batista D, Courkoutsakis NA, Oldfield EH (2005). Detection of adrenocorticotropin-secreting pituitary adenomas by magnetic resonance imaging in children and adolescents with Cushing disease. *Journal of Clinical Endocrinology and Metabolism*.

[21] Bonelli FS, Huston J, Carpenter PC, Erickson D, Young WF, Meyer FB (2000). Adrenocorticotropic hormone-dependent Cushing’s syndrome: sensitivity and specificity of inferior petrosal sinus sampling. *American Journal of Neuroradiology*.

[22] Lad SP, Patil CG, Laws ER, Katznelson L (2007). The role of inferior petrosal sinus sampling in the diagnostic localization of Cushing’s disease. *Neurosurgical Focus*.

[23] Oldfield EH, Doppman JL, Nieman LK (1991). Petrosal sinus sampling with and without corticotropin-releasing hormone for the differential diagnosis of Cushing’s syndrome. *The New England Journal of Medicine*.

[24] Kaltsas GA, Giannulis MG, Newell-Price JDC (1999). A critical analysis of the value of simultaneous inferior petrosal sinus sampling in Cushing’s disease and the occult ectopic adrenocorticotropin syndrome. *Journal of Clinical Endocrinology and Metabolism*.

[25] Scheithauer BW, Jaap AJ, Horvath E (2000). Clinically silent corticotroph tumors of the pituitary gland. *Neurosurgery*.

[26] Biller BMK, Grossman AB, Stewart PM (2008). Treatment of adrenocorticotropin-dependent cushing’s syndrome: a consensus statement. *Journal of Clinical Endocrinology and Metabolism*.

[27] Aghi MK (2008). Management of recurrent and refractory Cushing disease. *Nature Clinical Practice Endocrinology and Metabolism*.

[28] Blevins LS, Sanai N, Kunwar S, Devin JK (2009). An approach to the management of patients with residual Cushing’s disease. *Journal of Neuro-Oncology*.

[29] Lindsay JR, Nansel T, Baid S, Gumowski J, Nieman LK (2006). Long-term impaired quality of life in Cushing’s syndrome despite initial improvement after surgical remission. *Journal of Clinical Endocrinology and Metabolism*.

[30] Sonino N, Bonnini S, Fallo F, Boscaro M, Fava GA (2006). Personality characteristics and quality of life in patients treated for Cushing’s syndrome. *Clinical Endocrinology*.

[31] Laws ER, Reitmeyer M, Thapar K, Vance ML (2002). Cushing’s disease resulting from pituitary corticotrophic microadenoma: treatment results from transsphenoidal microsurgery and gamma knife radiosurgery. *Neurochirurgie*.

[32] Chen JCT, Amar AP, Choi S, Singer P, Couldwell WT, Weiss MH (2003). Transsphenoidal microsurgical treatment of Cushing disease: postoperative assessment of surgical efficacy by application of an overnight low-dose dexamethasone suppression test. *Journal of Neurosurgery*.

[33] Hammer GD, Tyrrell JB, Lamborn KR (2004). Transsphenoidal microsurgery for Cushing’s disease: initial outcome and long-term results. *Journal of Clinical Endocrinology and Metabolism*.

[34] Jagannathan J, Smith R, DeVroom HL (2009). Outcome of using the histological pseudocapsule as a surgical capsule in Cushing disease: clinical article. *Journal of Neurosurgery*.

[35] Meij BP, Lopes MBS, Vance ML, Thorner MO, Laws ER (2000). Double pituitary lesions in three patients with Cushing’s disease. *Pituitary*.

[36] Esposito F, Dusick JR, Cohan P (2006). Clinical review: early morning cortisol levels as a predictor of remission after transsphenoidal surgery for Cushing’s disease. *Journal of Clinical Endocrinology and Metabolism*.

[37] Nakane T, Kuwayama A, Watanabe M (1987). Long term results of transsphenoidal adenomectomy in patients with Cushing’s disease. *Neurosurgery*.

[38] Locatelli M, Vance ML, Laws ER (2005). Clinical review: the strategy of immediate reoperation for transsphenoidal surgery for Cushing’s disease. *Journal of Clinical Endocrinology and Metabolism*.

[39] Valassi E, Biller BMK, Swearingen B (2010). Delayed remission after transsphenoidal surgery in patients with cushing’s disease. *Journal of Clinical Endocrinology and Metabolism*.

[40] Engelhardt D, Weber MM (1994). Therapy of Cushing’s syndrome with steroid biosynthesis inhibitors. *Journal of Steroid Biochemistry and Molecular Biology*.

[41] Gross BA, Mindea SA, Pick AJ, Chandler JP, Batjer HH (2007). Medical management of Cushing disease. *Neurosurgical Focus*.

[42] Basina M, Liu H, Hoffman AR, Feldman D (2012). Successful long-term treatment of Cushings disease with mifepristone (RU486). *Endocrine Practice*.

[43] Minniti G, Brada M (2007). Radiotherapy and radiosurgery for Cushing’s disease. *Arquivos Brasileiros de Endocrinologia e Metabologia*.

[44] Jagannathan J, Sheehan JP, Pouratian N, Laws ER, Steiner L, Vance ML (2007). Gamma Knife surgery for Cushing’s disease. *Journal of Neurosurgery*.

[45] Jagannathan J, Yen CP, Pouratian N, Laws ER, Sheehan JP (2009). Stereotactic radiosurgery for pituitary adenomas: a comprehensive review of indications, techniques and long-term results using the Gamma Knife. *Journal of Neuro-Oncology*.

[46] Thompson SK, Hayman AV, Ludlam WH, Deveney CW, Loriaux DL, Sheppard BC (2007). Improved quality of life after bilateral laparoscopic adrenalectomy for cushing’s disease: a 10-year experience. *Annals of Surgery*.

